# Simultaneous Kinetics of Selenite Oxidation and Sorption on δ-MnO_2_ in Stirred-Flow Reactors

**DOI:** 10.3390/ijerph18062902

**Published:** 2021-03-12

**Authors:** Zheyong Li, Yajun Yuan, Lin Ma, Yihui Zhang, Hongwei Jiang, Jiqiang He, Yifan Hu, Shoushu Yuan, Matthew Ginder-Vogel, Shuxin Tu

**Affiliations:** 1 College of Resources and Environment, Huazhong Agricultural University, Wuhan 430070, China; lizheyong@webmail.hzau.edu.cn (Z.L.); zhangyihui@webmail.hzau.edu.cn (Y.Z.); hwjiang1997@163.com (H.J.); hjq951207@163.com (J.H.); xzy11@webmail.hzau.edu.cn (Y.H.); yuan.ss@foxmail.com (S.Y.); 2 Hubei Research Centre for Environment Pollution and Remediation, Wuhan 430070, China; 3 School of Resources and Environmental Engineering, Wuhan University of Technology, Wuhan 430070, China; yuanxm1225@163.com; 4 Department of Environmental Engineering Design, Hubei Urban Construction Design Institute Co., Ltd., Wuhan 430051, China; 5 CAS Key Laboratory of Aquatic Botany and Watershed Ecology, Wuhan Botanical Garden, Chinese Academy of Sciences, Wuhan 430074, China; malin@wbgcas.cn; 6 Chenzhou Dongjiang Lake Water Environmental Protection Bureau, Chenzhou 423000, China; 7 Department of Civil and Environmental Engineering, University of Wisconsin-Madison, Madison, WI 53706, USA; matt.ginder-vogel@wisc.edu

**Keywords:** selenium, oxidation, adsorption, manganese oxide, simultaneous kinetics

## Abstract

Selenium (Se) is an essential and crucial micronutrient for humans and animals, but excessive Se brings negativity and toxicity. The adsorption and oxidation of Se(IV) on Mn-oxide surfaces are important processes for understanding the geochemical fate of Se and developing engineered remediation strategies. In this study, the characterization of simultaneous adsorption, oxidation, and desorption of Se(IV) on δ-MnO_2_ mineral was carried out using stirred-flow reactors. About 9.5% to 25.3% of Se(IV) was oxidized to Se(VI) in the stirred-flow system in a continuous and slow process, with the kinetic rate constant k of 0.032 h^−1^, which was significantly higher than the apparent rate constant of 0.0014 h^−1^ obtained by the quasi-level kinetic fit of the batch method. The oxidation reaction was driven by proton concentration, and its rate also depended on the Se(IV) influent concentration, flow rate, and δ-MnO_2_ dosage. During the reaction of Se(IV) and δ-MnO_2_, Mn(II) was produced and adsorbed strongly on Mn oxide surfaces, which was evidenced by the total reflectance Fourier transform infrared (ATR-FTIR) results. The X-ray photoelectron spectroscopy (XPS) data indicated that the reaction of Se(VI) on δ-MnO_2_ produced Mn(III) as the main product. These results contribute to a deeper understanding of the interface chemical process of Se(IV) with δ-MnO_2_ in the environment.

## 1. Introduction

Selenium (Se) is a redox-sensitive element that could be both deficient and excessive in the environment [1,2]. In a natural environment, Se is highly irregularly distributed, with Se content of 0.01–2 mg kg^−1^ in most soils and over 1200 mg kg^−1^ in Se-rich or Se-contaminated soils [3,4]. Geogenic and anthropogenic activities lead to vast Se pollutions in soil, consequently affecting its bioaccumulation in the food chain of the peripheral regions [5,6]. As Se cycling and transport in the near-surface soil zone leads to detailed interaction between Se in the environment and the health of living things, the study of the environmental behavior of Se in soil has received tremendous attention in recent decades.

In the soil environment, the mobility of Se is strongly governed by its speciation [7]. The main forms of Se in the environment are selenite (Se(IV)) and selenate (Se(VI)), and these are more stable and more common relative to other inorganic reducing and organic states of Se [8,9]. Meanwhile, Se(IV) and Se(VI) mainly exist in the form of inorganic oxygenated anions, and are prone to binding with metal oxides in soil. Furthermore, Se(IV) is more toxic and pervasive compared to Se(VI) [10]. Therefore, the process of Se interaction with metal oxides in soils and sediments strongly affects the solubility, mobility, and bioavailability of Se [11].

The interaction of Se with a natural mineral adsorbent is crucial for the description and prediction of the immobilization processes of Se at solid/liquid interfaces [12]. Adsorbents employed in previous studies include manganese oxides, iron oxide, aluminum oxide, zero-valent iron, montmorillonite, and their composites [13,14,15,16,17,18,19]. Among these adsorbents, manganese (Mn) oxides are recognized as a potent oxidant with high reactivity and large specific surface area that would influence the migration and fate of Se greatly. To date, some attempts have been made to investigate the interaction between Se and Mn oxides [20,21,22,23,24,25]. However, these studies are based on a static batch treatment method, and the dynamic process has not been extensively understood.

The stirred flow method is a novel method for studying the chemical dynamics of soils. It provides a better simulation of the open flow state of the actual soils, greatly reducing the inverse reactions and side reactions caused by product accumulation, and therefore more closely resembles the chemical kinetic process [26]. The ion exchange, adsorption, and analytical kinetics were often simulated by stirred-flow methods in soil and environmental chemistry studies [26,27,28]. A stirred-flow method has also been reported in the study of As (III), Sb(III), and other redox systems with manganese minerals, with a great advantage of conducting adsorption, oxidation, and desorption in one system [29,30,31]. Hence, it is effective and imperative to undertake a quantitative description of Se(IV) and Mn oxide reactions by the stirred-flow method.

In this study, we investigated the dynamic process of Se(IV) oxidation and adsorption on manganese oxide via a stirred-flow method. We hypothesized that selenium simultaneous adsorption and oxidation on manganese oxide occurs with different speed limiting conditions. The objectives of the study were as follows: (1) to investigate the simultaneous kinetics process of adsorption and oxidation of Se(IV) on manganese oxide by the stirred-flow method; (2) to explore selenium adsorption and oxidation affected by flow rate, initial Se(IV) concentration, solution pH, and δ-MnO2 dosage; and (3) to reveal the mechanisms of simultaneous Se oxidation and adsorption by manganese oxide through X-ray powder diffraction (XRD), X-ray photoelectron spectroscopy (XPS), and attenuated total reflectance Fourier transform infrared (ATR-FTIR).

## 2. Materials and methods

### 2.1. Materials

δ-MnO_2_, a widely used synthetic form of birnessite-like minerals that is both reactive and abundant in the environment [32], was prepared by dissolving 11.29 g of Mn(NO_3_)_2_∙4H_2_O in 100 mL of ultrapure water, followed by dropwise addition to a 100 mL solution containing 2.4 g of NaOH and 4.74 g of KMnO_4_ and stirring overnight. The resulting slurry was centrifuged and washed five times in ultrapure water. The solids were then suspended in ultrapure water, stored at 4 °C, and used within two weeks. Gravimetric analysis was introduced to determine the slurry concentration.

The combined material characterized by X-ray diffraction (XRD; Bruker D8 Advance Germany, Mo Kα source; λ = 0.7093 Å) (Bruker Co., Karlsruhe, Germany) showed that δ-MnO_2_ was the only crystalline phase produced (Figure S1). The XRD spectra of δ-MnO_2_ revealed that the dominant peak was located at 37° and 66°, suggesting hexagonal symmetry and that δ-MnO_2_ was the only product of the synthesis.

Scanning electron microscope (SEM VEGA 3 XMU, Tescan, Shanghai, China) images proved that pristine δ-MnO_2_ particles were poorly crystalline (Figure S2). X-ray photoelectron spectroscopy (XPS) data showed that the valence state of δ-MnO_2_ was 3.30 valence units, with percentages of Mn(IV), Mn(III), and Mn(II) at 54%, 22%, and 24%, respectively. The average valence state of Mn in the δ-MnO_2_ structure determined by oxalate titration was 3.43 ± 0.12 v.u, which was corroborated by XPS values.

Sodium selenite (ACS 100%), sodium selenate (ACS 100%), calcium chloride dihydrate (ACS, 100%), sodium acetate trihydrate (ACS, 100%), sodium nitrate (ACS, 100%), manganese(II) nitrate tetrahydate (analytical grade), and potassium permanganate (ACS) were purchased from Fisher Chemical.

### 2.2. Kinetics Experiments

A batch treatment experiment and a stirred-flow experiment were used to characterize the kinetic process of Se(IV) oxidation and adsorption on δ-MnO_2_.

#### 2.2.1. Batch Experiments

The batch experiment was conducted to investigate the effects of pH (4.0–7.0) and Se(IV) concentrations (50–200 μM) on the oxidation and adsorption reaction on δ-MnO_2_ (0.5 g L^−1^), with 10 mM of NaNO_3_ as the background electrolyte. The solution pH was adjusted using 0.1 M of HCl and 0.1 M of NaOH, and the suspensions were shaken at 25 °C. After 700 h, the supernatants were obtained by centrifugation and filtration through 0.22 μm filter membranes for the determination of Se(IV). Solids were recovered at the end of the reaction. The sample was washed three times in deionized water, dried at room temperature, and used for characterization by XRD (data not shown) and XPS.

#### 2.2.2. Stirred-Flow Experiments

The stirred-flow kinetic reactor was assembled according to Balgooyen [28]. Briefly, 15 mL of 5–20 g L^−1^ δ-MnO_2_ suspension was deposited in the 20 mL reaction chamber with a magnetic stirring bar prior to the experiment. The electrolyte solutions containing 10 mM of NaNO_3_ and 10 mM of acetate at pH values of 4.0, 5.0, 6.0, and 7.0 were each introduced into the reactor by a peristaltic pump at a steady rate for 2 h at 25 °C. A 25 mm diameter filter membrane with a 0.45 μm pore size was used to retain the solid in the chamber. Subsequently, a solution containing 50–200 μM of Se(IV), 10 mM of NaNO_3_, and 10 mM of acetate at pH 4.0, 5.0, 6.0, and 7.0 was pumped into the reaction chamber from a reservoir at flow rates of 0.5, 1, and 2 mL min^−1^, which were employed to meet the mobility of the soil environment [33]. Control tests were conducted by introducing Se(IV) without δ-MnO_2_ in the chamber, and the results are shown in Figure S4. The desorption experiment was conducted afterward, in which the blank solution containing 10 mM of NaH_2_PO_4_, 10 mM of CaCl_2_, or 10 mM of NaNO_3_ and 10 mM of acetate at pH 4.0 alone was pumped into the reaction chamber. The effluent was collected during the entire adsorption/desorption process by a fraction collector to determine the effluent concentrations of Se(IV), Se(VI), and Mn(II).

### 2.3. Analysis Methods

#### 2.3.1. Chemical Analysis

Se(IV) and Se(VI) were determined using hydride generation atomic fluorescence spectroscopy (HG-AFS) (AFS-8220, Beijing Titan Instrument Co., Ltd., Beijing, China) [34]. The hydrides were prepared using a mixture of 2% KBH_4_ (0.5% KOH preparation) and 5% HCl. The reducing agent used 5% ascorbic acid and 5% thiourea mixed with 2 M of HCl. The solution sample was measured in two parts: the selenium concentration was measured directly as the Se(VI) concentration and after acid digestion as the total Se concentration (Se_tot_). The Mn content was determined by flame atomic absorption spectroscopy (240FSAA, Agilent, Palo Alto, CA, USA). Each test was performed in triplicate, and the average was taken as the result.

#### 2.3.2. XPS Determination

XPS was performed using a Thermal Scientifics K-Alpha XPS system (ESCALab-220i-XL, Waltham, MA, USA) with an Al Kα X-ray source. The test conditions were Al target, X-ray analysis, and vacuum 2 × 10^−7^ Pa. The full XPS spectrum of the sample and the narrow zone spectrum of each element were recorded, and the spectra were separated by multipeak Gaussian fit using Avantage software (Waltham, MA USA). C1s peaks were collected as an inner standard calibration peak at 284.7 eV. The energy spectra of Mn3p and Se3p orbital were determined and analyzed to quantify the oxidation state of Mn and Se.

#### 2.3.3. ATR-FTIR Spectra Determination

The reaction of Se(IV) with δ-MnO_2_ was determined using a BrukerEqulnox55 FTIR spectrophotometer (Bruker Co., Karlsruhe, Germany) with a diamond ATR crystal. A δ-MnO_2_ film was obtained by dropping 1 mL of 1 g L^−1^ δ-MnO_2_ solution into a stainless steel reactor and drying under an N_2_ atmosphere. The δ-MnO_2_ film was washed with background solution (10 mM of NaNO_3_) at a flow rate of 1 mL L^−1^ into the reaction chamber, and then the reaction was performed using 100 μM of Na_2_SeO_3_ solution (pH = 4) instead of background electrolyte for 6 h. The spectral data were collected, and the difference spectra were calculated and analyzed using OMNIC 8.0 software (Thermo Nicolet, Waltham, MA, USA).

### 2.4. Data Statistic Methods

#### 2.4.1. Model Fitting for Adsorption/Desorption Kinetics

In batch reactors, Se(IV) adsorption and oxidation were calculated by the pseudo first-order kinetic model:

(1) $$Q_{t}=Q_{e}\left(1-e^{-k_{1}t}\right)$$

where Q_t_ (mg g^−1^) is the amount of Se(IV) adsorbed or oxidized at time t, Q_e_ is the amount of Se(IV) adsorbed or oxidized at equilibrium, and k_1_ (h^−1^) is the rate constant of the pseudo first-order kinetic equation of the reaction.

The Langmuir model described in Equation (2) enables the determination of the maximum adsorption capacity of Se(IV) on δ-MnO_2_ [35]:

(2) $${\;Q}_{t}={\mathrm{Ck}}_{2}Q_{m}/\left(1+{\mathrm{Ck}}_{2}\right)\;$$

where Q_m_ (μmol g^−1^) is a constant related to the maximum amount of the adsorption, C (μM) is the concentration of sorbate at equilibrium, and k_2_ is the constant related to adsorption affinity.

In stirred-flow reactors, Se(IV) oxidation kinetics were calculated using Equation (3) described by [36]:

(3) $$C_{t}{=C}_{0}(\frac{F}{F+k_{3}V}\cdot e^{-\left(\frac{F}{V}+k_{3}\right)\cdot t}+\frac{k_{3}V}{F+k_{3}V}{)-C}_{0}\cdot e^{-\frac{F\cdot t}{V}}\;$$

where C_t_ (μmol g^−1^) is the amount of Se(VI) in the effluent, C_0_ is the Se(IV) introduction concentration, F is the flow rate, V is the reactor volume, and k_3_ is the oxidation rate of Se(IV) on δ-MnO_2_.

#### 2.4.2. Data Statistics

All of the adsorption and oxidation experiments were performed in triplicate, and experimental blanks were run in parallel. Sorption and desorption isotherm data were obtained by Origin2018 (Microsoft, Redmond, WA, USA). X-PS data were analyzed by Avantage and Mn2p best fit using Mn(II), Mn(III), and Mn(IV) parameters derived from MnO, MnOOH, and MnO_2_, respectively [37]. ATR-FTIR was analyzed using OMNIC 8.0 software to calculate the difference spectra.

## 3. Results

### 3.1. Kinetics of Sorption and Oxidation in the Batch Experiment

**Figure 1:**
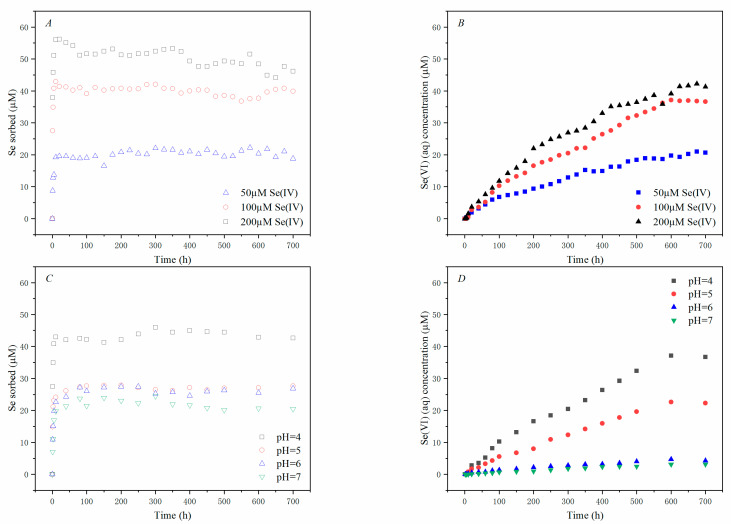
The effects of initial concentrations of Se(IV) and solution pH on the adsorption and oxidation of Se(IV) by δ-MnO_2_ in batch experiments. (**A**) Se(IV) adsorbed; (**B**) Se(VI) (aq) concentration with initial Se(IV) concentrations of 50, 100, and 200 μmol L^−1^; δ-MnO_2_ dosages of 10 g L^−1^ in a pH 4 acetate buffer; (**C**) Se adsorbed; (**D**) Se(VI) (aq) concentration with a pH of 4, 5, 6, and 7, initial Se(IV) concentrations of 100 μmol L^−1^, and δ-MnO_2_ dosages of 10 g L^−1^.

The batch experiment was conducted to investigate the effects of Se(IV) sorption and oxidation on δ-MnO_2_ under different initial Se(IV) concentrations and solution pH conditions (Figure 1). The results showed that both adsorption and oxidation of Se(IV) on δ-MnO_2_ did occur, and the adsorption of Se(IV) on δ-MnO_2_ happened rapidly, increasing 19.6–56.2% within 15–30 min, while Se(VI) (aq) only appeared 5 h after Se(IV) was introduced, indicating a relatively slow oxidation process.

The kinetics data were generally well-fitted with the second-order kinetics (Table 1) for the Se(IV) adsorption process on δ-MnO_2_ (R^2^ = 0.829–0.994). The highest rate of Se(IV) adsorption could be 0.926 h^−1^, with an equilibrium concentration of 43.1 µM when 100 µM of Se(IV) reacted with 10 g of L^−1^ δ-MnO_2_ (pH = 4) (Figure 1A,C). Adsorption isothermal curves at different initial Se(IV) concentrations (10 µM–200 µM) were fitted using the Langmuir model with an R^2^ of 0.996, yielding a capacity of 76.13 µmol g^−1^ for Se(IV) reacted with δ-MnO_2_ (Figure S3). Se(VI), the oxidation product, was produced continuously at a rate of 0.0014 h^−1^ after its appearance, and the reaction continued for 700 h, at which about 61.6% of Se(IV) was oxidized to Se(VI).

The adsorption rate of Se(IV) decreased, but the amount of oxidation increased with increasing initial concentrations of Se(IV) (Figure 1A,B). When the initial concentration of Se(IV) increased from 50 to 200 μM, the adsorption rate decreased by 68.6%, and the amount of oxidation increased by 2.4 times.

The elevated solution pH significantly reduced Se(IV) adsorption and oxidation (Figure 1C,D). The highest Se(IV) adsorption (43.1 μmol) was at pH = 4. As the pH increased to 5, 6, and 7, the adsorption decreased by 48.5%, 42.8%, and 56.1%, respectively. The rate of oxidation of Se(IV) by δ-MnO_2_ followed the same trend with increasing pH, for the concentration of the oxidation product Se(VI) decreased more significantly at 36.4%, 86.6%, and 90.7%, respectively.

### 3.2. Adsorption and Oxidation Kinetics in the Stirred-Flow Experiment

**Figure 2:**
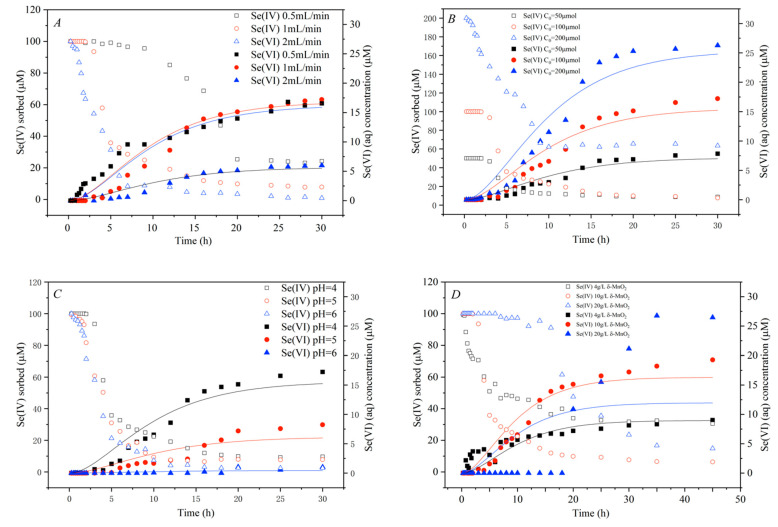
The amount of Se(IV) adsorbed and Se(VI) in the effluent, as well as oxidation kinetics of a stirred-flow experiment reacting at the flow rate of (**A**) 0.5, 1, and 2 mL L^−1^ of (**B**) 50, 100, and 200 μmol L^−1^ Se(IV) (C_0_) with (**C**) a pH 4, 5, and 6, and with (**D**) 4, 10, and 20 g L^−1^ δ-MnO_2_. Symbols are experimental data, and lines are model calculations.

The stirred-flow reactor experiment was conducted with varying Se(IV) initial concentrations (50–200 μM), δ-MnO2 dosages (4–20 g L^−1^), flow rates (0.5–2 mL min^−1^), and solution pH (4–7) to determine their effects on the reaction process and oxidation kinetics. The Se(IV) adsorbed was calculated by subtracting from the Se(IV) in the influent and the total Se in the effluent. Initially, as shown in Figure 2, Se(IV) was completely adsorbed by δ-MnO_2_, emerging as the first plateau of Se(IV) adsorption. By this time, the oxidation product Se(VI) was below detection or in a very low concentration in the effluent. As the Se(IV) adsorption amount decreased due to the reaction sites on δ-MnO_2_ being occupied, Se(VI) concentration continually increased in the effluent. After the manganese oxide became less reactive, the amount of Se(IV) adsorbed reached a second plateau, while the Se(VI) concentration was close to unchanged.

#### 3.2.1. Effects of Flow Rate

Flow rate had an obvious impact on the processes of Se(IV) adsorption and oxidation, because it greatly influenced the injection rate, retention time, and desorption rate of Se(IV) (Figure 2A). By raising the flow rate, the amount of Se(IV) adsorbed decreased remarkably. The first plateau duration of Se(IV) adsorption also decreased gradually from 10 h to 3 h and 0.5 h with the increase of flow rate from 0.5 mL min^−1^ to 1 and 2 mL min^−1^, after which the rate of decrease of Se adsorption accelerated.

In the effluent, the appearance of the oxidation product Se(VI) was gradually delayed as the flow rate increased, while Se(VI) concentration was much lower at the flow rate of 2 mL min^−1^ than at 0.5, and 1 mL min^−1^ after 15 h of reaction (Figure 2A). The proportions of Se(IV) oxidized (Se(VI) effluent/Se(IV) influent) were 2.3%, 6.7%, and 10.8% as the flow rates were 0.5, 1, and 2 mL min^−1^, respectively, at 8 h of reaction. At flow rates of 1 and 2 mL min^−1^, the oxidation rate was close, reaching 2.9 and 3.2 times the oxidation rate at 0.5 mL L^−1^ (Table 2).

#### 3.2.2. Effect of Initial Se(IV) Concentrations

Se(IV) adsorption varied with the initial Se(IV) concentration, with higher concentrations of Se(IV) leading to higher Se(IV) adsorption (Figure 2B). The first plateau duration of Se(IV) adsorption decreased from 3.5 h to 3 h and 0.75 h, with the Se(IV) injection concentration increasing from 50 μM to 100 and 200 μM, after which the decrement of Se(IV) adsorption accelerated.

The concentrations of the oxidation product Se(VI) in the effluent increased with the initial concentration of Se(IV) (Figure 2B). However, the proportion of Se(IV) oxidized (Se(VI) effluent/Se(IV) influent) was 15.6%, 17.2%, and 13.1% at 30 h of reaction, respectively, with no significant change. Likewise, the oxidation rate did not change significantly (Table 2).

#### 3.2.3. Effect of Solution pH

Solution pH had a noticeable effect on Se(IV) adsorption, which decreased dramatically at a higher pH (Figure 2C). The first plateau duration of Se(IV) adsorption decreased from 3 h to 1.75 h and 1 h as the solution pH increased from 4 to 5 and 6, after which the decrement of Se adsorption accelerated.

The oxidation capacity of δ-MnO_2_ decreased as the pH increased, and the oxidation reaction almost ceased when the pH came to 6. The proportion of Se(IV) oxidized (Se(VI) effluent/Se(IV) influent) was 17.2%, 8.22%, and 1.0% at 30 h of reaction, respectively (Figure 2C). The kinetic fit was better at a pH of 4 and 5, and the oxidation rate decreased by 62.5% as the pH increased from 4 to 5 (Table 2).

#### 3.2.4. Effect of δ-MnO_2_ Injection

As expected, enhancing the dosage of δ-MnO_2_ significantly increased the adsorption of Se(IV) (Figure 2D). The first plateau duration of Se(IV) adsorption increased from 0 h to 3 h and 15 h with increasing δ-MnO_2_ injection from 4 to 10 and 20 g L^−1^, after which the rate of decline of Se adsorption slowed down.

The concentrations of the oxidation product Se(VI) in the effluent decreased with increasing δ-MnO_2_ input during the initial phase of the reaction (before 10 h), while the concentrations of Se(VI) increased with increasing δ-MnO_2_ input after 20 h of reaction. In this case, the change in Se(VI) concentration before 10 h and after 20 h may be attributed to the excess Se(IV) acting to promote the release of the oxidation product Se(VI). The proportions of Se(IV) oxidized (Se(VI) effluent/Se(IV) influent) were 9.0%, 19.2%, and 26.4% after 45 h of reaction, respectively (Figure 2D). The Se(IV) oxidation rate nearly doubled after the δ-MnO_2_ input was increased from 4 g L^−1^ to 10 g L^−1^ (Table 2). At a 20 g L^−1^ input of the manganese oxide, the fit was poor due to the greatly increased Se adsorption, which did not match the model assumptions.

### 3.3. Desorption Kinetics of Se(IV) and Se(VI) in the Stirred-Flow Experiment

Desorption experiments were performed in a stirred-flow reactor by stopping the reaction between Se(IV) and δ-MnO_2_ after 24 h, and simultaneously beginning desorption by NaH_2_PO_4_, CaCl_2_, or background electrolyte alone. NaH_2_PO_4_ represents an anion chemically similar to the selenium anion, and was expected to desorb Se(IV). CaCl_2_ represents a cation capable of reacting with the active site of manganese oxide, and had the potential to desorb Mn(II), which was not detected in the solution of the reactions in both batch and stirred-flow experiments. Background electrolyte (acetic buffer and NaNO_3_) was likely to react weakly with δ-MnO_2_ reactive sites, and should exhibit some desorption potential.

**Figure 3:**
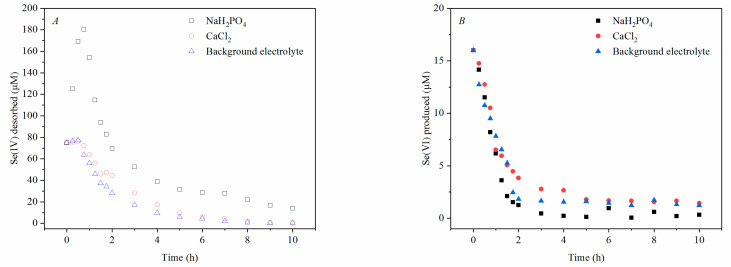
Se(IV) desorbed (**A**) and Se(VI) produced (**B**) by NaH_2_PO_4_, CaCl_2_, and background electrolyte after Se(IV) oxidation by δ-MnO_2_ for 24 h.

As shown in Figure 3A, NaH_2_PO_4_ was more effective in the desorption of Se(IV) than CaCl_2_ and the background electrolyte. By integrating the Se(IV) adsorption concentration with time, the amounts of Se(VI) desorbed by NaH_2_PO_4_, CaCl_2_, and background electrolyte calculated to be 447.5 μmol, 200.1 μmol, and 165.7 μmol, respectively.

However, compared to CaCl_2_ and background electrolyte, less Se(VI) was produced, and its concentration fell faster after NaH_2_PO_4_ was introduced. The amounts of Se(VI) produced after adding NaH_2_PO_4_, CaCl_2_, and background electrolyte were 17.4 μmol, 33.9 μmol, and 28.1 μmol, respectively. Separate Se(VI) adsorption tests showed that Se(VI) did not adsorb to the surface of δ-MnO_2_ (Figure S4). The Se(VI) produced in the desorption test should come from the oxidation process.

### 3.4. Analysis of XPS Spectra

Se(IV) adsorption samples in batch experiments were collected for XPS spectra to determine the valence states of Mn and adsorbed Se on δ-MnO_2_. The results showed that the predominant form of Mn on δ-MnO_2_ was Mn(III), which was up to 50.33%, and Se(IV) was the only form of adsorbed Se detected on δ-MnO_2_ after 700 h of reaction with Se(IV).

**Figure 4:**
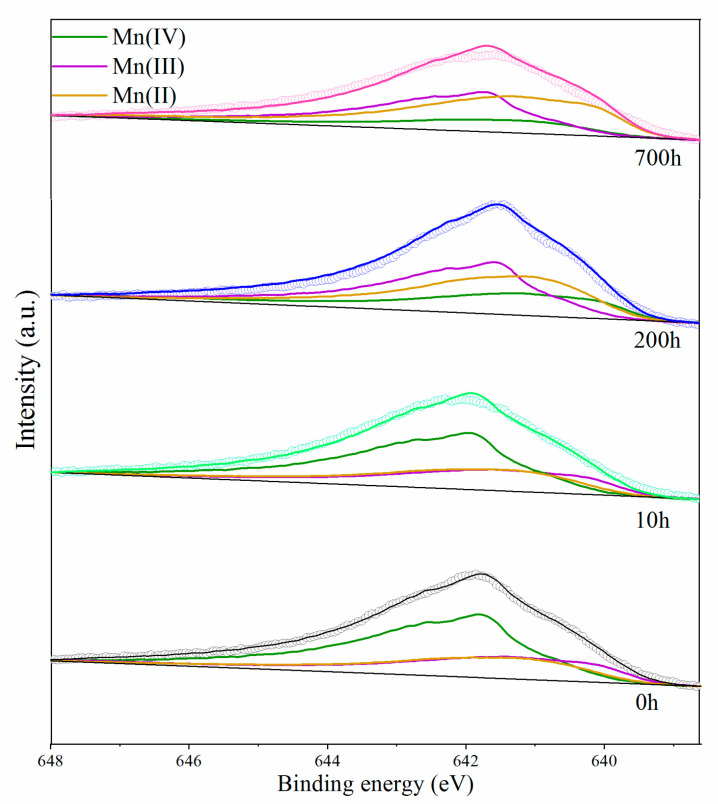
δ-MnO_2_ reacted with fitted Mn2p3/2 spectrum of δ-MnO_2_ after reaction with 100 μM of Se(IV) at a pH of 4 and 25 °C for 0 h, 10 h, 200 h, and 700 h.

The spectral peaks were fitted by a nonlinear fitting method to the spectral peaks according to the standard spectra to obtain the proportional relationship between the different chemical states (Figure 4). Mn2p spectra was used to determine changes in the δ-MnO_2_ oxidation state during the Se(IV) reactions. The Mn in pristine δ-MnO_2_ exhibited an average oxidation state at 3.30. After reacting with Se(IV), the average Mn oxidation states ranged from 3.27 to 2.98, which coincided with the decrease in the Mn oxidation state from Mn(IV) to Mn(III/II) (Table 3). After 700 h of reaction, the percentages of Mn(IV) decreased significantly from 54.22% to 26.59%, while Mn(III) and Mn(II) increased from 21.76% to 47.33%, and 24.02% to 25.08%, respectively. In this case, the Mn(III) content continued to increase during the 700 h of reaction, and had a significant and continuous increment, while the Mn(II) content only increased slightly, which indicated that Mn(II) was not the major product. Besides, the peak of Se3p in the range of 164.60 did not change with reaction time, and no significant Se(VI) adsorption peak appeared (Figure S5).

### 3.5. Analysis of ATR-FTIR Spectra

**Figure 5:**
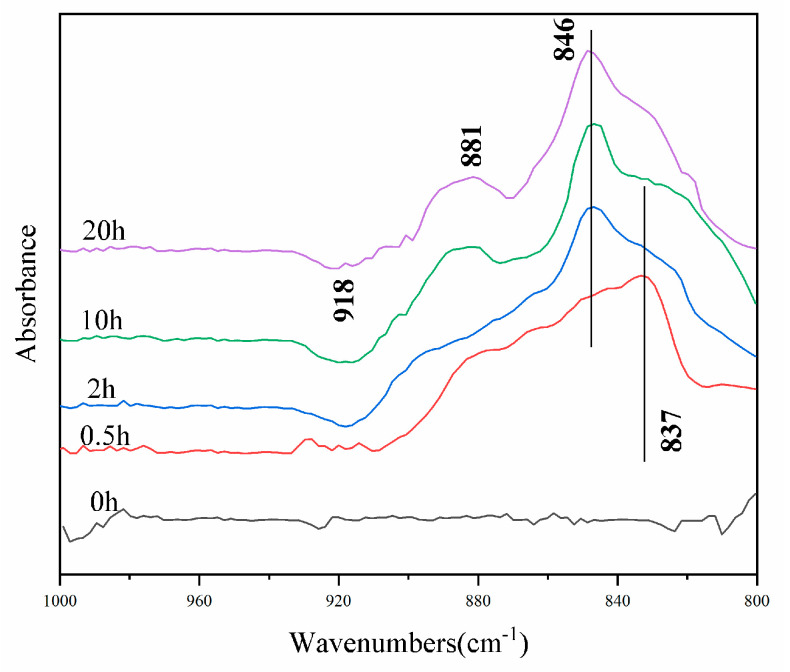
The real-time ATR-FTIR spectra of the in situ flow experiment of 100 μmol L^−1^ Se(IV) oxidation on δ-MnO_2_ at a pH of 4 and 25 °C.

The dynamic changes of the featured peaks of adsorbed Se species were presented in the real-time spectra of in situ Se(IV) oxidation on δ-MnO_2_ (Figure 5). The bands at 837 cm^−1^ were observed at the beginning of the reaction and the shift of 837 cm^−1^ to 846 cm^−1^, which could indicate the formation of a Se(IV) surface complex [38]. A new peak emerged at 881 cm^−1^, which was attributed to Se(VI) in the solution. No splitting or shifting of the peak indicated that Se(VI) was not adsorbed to δ-MnO_2_ [12]. After 2 h of reaction, the negative peak at 918 cm^−1^ began to emerge, and became progressively pronounced with time. It was probably because the Mn(II) generated by the oxidation reaction occupied the vacancy sites on the surface of the manganese oxide [31].

## 4. Discussion

### 4.1. Adsorption Performance

#### 4.1.1. Se(VI) Adsorption

There was no distinct Se(VI) adsorbed on δ-MnO_2_ under different conditions in batch or stirred-flow experiments (Figure S3). XPS results agreed with this contention, which revealed that no Se(VI) was detected on the δ-MnO_2_ surface (Figure S5). Similar results from previous studies have reported that Se(VI) can only be slightly adsorbed on Mn oxide at a pH lower than 4 [14,39].

#### 4.1.2. Se(IV) Adsorption

The sorption experiments and XPS results indicated that only Se(IV) was adsorbed on δ-MnO_2_, and the isotherm was well-fitted using a Langmuir model (R^2^ = 0.996), which yielded the maximum sorption capacity of 95.06 μmol g^−1^ for Se(IV) (Table 1). It has been reported that when As(V) and Sb(V) were the main species adsorbed on δ-MnO_2_, they had maximum adsorption capacities of 213.2 and 140.8 μmol g^−1^, respectively [40]. Another study observed the maximum adsorption capacities of Sb(III) and Sb(V) on δ-MnO_2_ to be 834.2 and 342.0 μmol g^−1^, respectively [31]. These results indicated that the adsorption capacity and rate of Se(IV) on δ-MnO_2_ were lower than those of As and Sb.

Se(IV) adsorption follows pseudo first-order kinetics during the 700 h batch experiments, and the rates of adsorption were critically influenced by Se(IV) initial concentration and solution pH (Table 1 and Figure 1). Se(IV) has a higher adsorption rate on δ-MnO_2_ at a lower pH. A similar relationship between Se(IV) adsorption and pH was observed in the investigation of Se(IV) adsorption by iron and silicon oxides [41]. At a pH of 5, the reaction rate constant of Se(IV) was 0.765 h^−1^ when 100 μM of Se(IV) reacted with δ-MnO_2_. Wang et al. [40] reported that the depletion rates of As(V) and Sb(V) were 11.28 h^−1^ and 175.3 h^−1^ under similar conditions, respectively, which were much higher than those for Se(IV). However, the Se(IV) reaction rate turned to 0.330 h^−1^ at a pH of 7, while As(V) and Sb(V) were 0.368 h^−1^ and 0.213 h^−1^, respectively [40], which were fairly comparable. These results indicate that the effect of solution pH on Se adsorption was not as strong as its effect on As and Sb on δ-MnO_2_. We can conclude likewise that, although raising solution pH has a noticeable decrement on Se(IV) adsorption on δ-MnO_2_, Mn oxides have the ability to adsorb Se(IV) even in a neutral pH. Furthermore, the reduction in Se(IV) initial concentration increased the solid-to-liquid ratio in the reactor, resulting in more effective adsorption sites emerging and leading to an increment of Se(IV) adsorption rate [42].

In a stirred-flow reactor, the solution pH had a similar effect on Se(IV) adsorption (Figure 2C). Lower pH accounts for a weaker electrostatic repulsion between Se(IV) and δ-MnO_2_, resulting in a higher sorption amount [20]. Increasing the flow rate and Se(IV) initial concentration led to a shorter initial Se adsorption plateau period and a faster decrease in adsorption (Figure 2A). The increase in the flow rate and initial concentration led to an increase in the amount of Se injected into the sample (Figure 3B). Feng et al. [43] reported that the As(III) concentration in the effluent increases under high As(III) loadings in the reaction with manganese oxide, which leads to a faster decrease in adsorption. Besides, the δ-MnO_2_ dosage also has a comparable effect, as it determines the number of reaction sites.

### 4.2. Se(IV) Oxidation on δ-MnO_2_

#### 4.2.1. Effects of Se(IV) Oxidation

In this study, solution pH and flow rate had a noticeable influence on Se(IV) oxidation, which indicated that proton concentration and the duration of Se(IV) retention affect the reaction more significantly (Figure 1 and Figure 2A,C). Although Se can adsorb at neutral pH, the oxidation reaction was almost terminated at a pH of 6 (Figure 2C). By increasing the solution pH, the Se(IV) oxidation ratio decreased obviously due to the oxidation reaction being largely driven by the proton concentration [44]. In lower flow rates, more oxidation product (Se(VI)) appeared sooner, as demonstrated [28]. However, Balgooyen et al. [28] also obtained that the redox plateau should be delayed in a higher flow rate because of the increase in reactants, and the ratio of moles of reactant consumption should be the same in a different flow rate. During Se(IV) oxidation by δ-MnO_2_ in the stirred-flow reactor, more Se(VI) came out at a lower flow rate before 8 h, while Se(VI) concentrations were close at the flow rates of 0.5 and 1 mL min^−1^ in a 30 h reaction (Figure 2A). This was probably because Se(IV) had a longer retention time in the lower flow rate, and the reaction was limited by the number of reaction sites on Mn oxides after 8 h. The results indicated that the oxidation process still takes a relatively long time to complete after Se(IV) adsorbed on δ-MnO_2_ [20].

#### 4.2.2. Comparison of Batch Experiments and Stirred-Flow Experiments

The oxidation rate in the stirred-flow test (0.032 h^−1^) was significantly faster than the results in the batch test (0.0014 h^−1^), indicating that the oxidation rate in a stirred-flow reactor was closer to the true oxidation rate, because it can effectively simulate the soil flow environment [26,45]. Scott et al. [20] obtained that the electron transfer rate was the main rate-limiting step of Se(IV) oxidation on Mn oxides. In this study, a higher oxidation rate was observed in the stirred-flow reactor, since it could release Se(VI) faster. If the electron transfer rate was the only step that limited the reaction, Se(VI) should be absent in the initial stages of reaction and become abundant in the late stages. However, Se(VI) came out at a steady rate in both batch and stirred-flow reactors, indicating that Se(VI) release also limited Se(IV) oxidation.

### 4.3. Reaction Mechanisms

#### 4.3.1. Adsorption Mechanisms

Our desorption experiments indicated that the adsorption of Se(IV) forms strong bonds on the surface of manganese oxides (Figure 3). The adsorption of phosphate was suggested to take place on the inner sphere, and about 70.0% of the adsorbed Se(IV) was desorbed by NaH_2_PO_4_ in the stirred-flow reactor, which corresponds to a report by K. Saeki et al. [39]. However, it cannot be concluded from the ATR-FTIR data whether the complexation of Se(IV) on manganese oxides is a mono or bidentate complex [38]. Foster et al. [22] observed that Se(IV) mainly forms complexes with Mn(II)O_6_ or Mn(III)O_6_ octahedral. However, Mn(II) was not simultaneously desorbed from Mn oxides with Se(IV) during the desorption (Figure 3), indicating that Se(IV) adsorption on δ-MnO_2_ might generate Se(IV)-O-Mn(III) as the main surface complex.

#### 4.3.2. Oxidation Mechanisms

During the Se(IV) reaction with δ-MnO_2_, the transfer of one or two electrons was responsible for the Se(IV) oxidation. Mn(IV) on the mineral surface was reduced by Se(IV), and the products were Mn(III) and Mn(II). The XPS spectra of Mn2p3/2 evidenced that Mn(II) was sporadically produced, while Mn(III) content increased remarkably in the 700 h reaction. Nesbitt et al. [21] proffered that Mn(III) did not oxidize Se(IV) to produce Mn(II), so the production of Mn(III) and Mn(II) were due to the one-electron and two-electron transfer reaction, respectively. Furthermore, Mn(II) may come from the disproportionation reaction of Mn(III) [46,47]. However, there was only a rare increase in Mn(II), the most likely source of which was the disproportionation reaction. This indicates that the two-electron transfer was not the primary reaction process for Se(IV) oxidation. Therefore, Mn(IV) oxidized Se(IV) mainly to produce Mn(III) and Se(VI), while Mn(III) produced Mn(II) and Mn(IV) through the disproportionation reaction.

The reactivity of Mn oxides is often reduced by the emergence of passivation when the valence variable element reacts with manganese oxides, leading to a deduction of the oxidation rate. For example, during the reaction of As(III) with δ-MnO_2_, the adsorption of products As(V) and Mn(II), the formation of Mn-As precipitation, and the formation of Mn(III) intermediate products lead to surface passivation of manganese oxides, and the reaction turns from efficient to inefficient [48]. Similar to As(III), the passivation of δ-MnO_2_ reacted with Sb(III) was attributed to the adsorption of Sb(V) and Mn(II), the formation of Mn-Sb precipitation, and the formation of Mn(III) intermediate products [31]. Precipitation of Cr(OH)_3_ on the surface decreases the oxidation efficiency of δ-MnO_2_ on Cr(III) [49]. Adsorption of U(VI) also results in decreased activity of δ-MnO_2_ [50].

However, in the case of Se(IV) reacted with δ-MnO_2_, the reaction did not reach equilibrium until 700 h after the batch method experiment, the product Se(VI) was still produced, and no surface passivation was observed with a significant decrease in the reaction rate. There may be several reasons for the absence of mineral passivation.

Firstly, Se(IV) oxidation products are difficult to adsorb on mineral surfaces. Se(VI) can hardly stay on the δ-MnO_2_ surface [22], and does not hinder the reactive sites due to high adsorption.

Meanwhile, the yield of Mn(II) was not significant, neither blocking the boundary sites nor complexing with Se(IV) to reduce oxidation efficiency. Mn(II) adsorption at cavity sites is more stable and difficult to resolve relative to the boundary sites [30]. In the desorption test, CaCl_2_ did not resolve Mn(II) from the post-reaction δ-MnO_2_ surface; the negative peak at 918 cm^−1^ in ATR-FTIR was attributed to Mn(II) adsorption to δ-MnO_2_ vacancy sites, extrapolating that the produced Mn(II) was adsorbed to δ-MnO_2_ vacancy sites, and was not enough to reach the edge sites.

Furthermore, the adsorption reaction of Se(IV) was relatively rapid, and the subsequent oxidation reaction was not affected by the large amount of Mn(III) produced. Although Mn(III) was the main product of the reaction and Mn(III) no longer appeared to involve itself in the oxidation of Se(IV) [21], the presence of Mn(III) did not affect the oxidation of Se(IV) already adsorbed in δ-MnO_2_, as the adsorption of Se(IV) essentially reached equilibrium in 30 min, and the subsequent oxidation reaction was so slow that the appearance of Mn(III) did not considerably affect the oxidation process of Se(IV), which was already adsorbed on δ-MnO_2_.

### 4.4. Environmental Implication

In the mineral water interface, the mobility of Se is not only heavily influenced by its chemical form, but it also plays an important role in the adsorption–desorption and oxidation–reduction reactions. For example, hydrotalcite has a good adsorption effect on both Se(IV) and Se(VI), but the presence of nitrate, sulfate, and phosphate will desorb some of Se(IV) and Se(VI), enhancing the mobility of Se [35]. In the present study, Se(IV) can be desorbed to a large extent from the surface of δ-MnO_2_ under three desorptives investigated, especially phosphate. When manganese oxides are involved at a natural pH, selenium is present, mainly in the form of Se(IV), and is immobilized on the manganese oxide surface, but oxidizes slowly. Although Se(VI) is more mobile in the environment [7], it exists in a low proportion in the water–manganese ore environment due to the restriction of oxidation rate. More importantly, the mobility of Se(IV) is greatly increased when there is ionic coexistence, especially of phosphate. These studies highlight the necessity to pay great attention to Se mobility when treating Se-contaminated sites with mineral sorbents, especially when there are abundant competing ions in the environment. At the same time, concerns about the transport and transformation of selenium in the environment are crucial for the health and safety of local animals and humans.

## 5. Conclusions

Diverse kinetic and equilibrium experiments were carried out to investigate the mechanisms of simultaneous Se(IV) adsorption and oxidation on δ-MnO_2_ in this study. The adsorption of Se(IV) on δ-MnO_2_ reached equilibrium in hours. ΔH^0^ and ΔG^0^ values indicated an endothermic adsorption reaction, and Se(IV) mainly formed an inner layer complex on δ-MnO_2_ due to ΔS^0^ < 0. Se(IV)-O-Mn(III) might be the main surface complex of Se(IV) adsorption on δ-MnO_2_. The oxidation was relatively slow, and two-electron transfer was the dominant process. Se(VI) and Mn(III) were the primary reaction products, which emerged at a steady rate. Both the electron transfer and oxidation product release rate should account for the slow rate of Se(IV) oxidation. No soluble Mn was detected in both oxidation and desorption experiments, and Mn(II) mainly adsorbed steadily on the surface of δ-MnO_2_. NaH_2_PO_4_ was an effective desorptive that could mobilize about 70.0% adsorbed Se(IV) on δ-MnO_2_. This study provides useful information on the mechanism of Se(IV) adsorption and oxidation on the surface of manganese oxides, generates ideas on the way for Se transformation in the environment, and aids in the protection and enhancement of health and safety for human and animal life.

## Figures and Tables

**Table 1:** The kinetic parameters of Se(IV) for isothermal adsorption and oxidation on δ-MnO_2_ in batch experiments.

Category	Factors		Parameters		
	pH	Se(IV) Initial Concentration (μM)	Q_max_^a^ (μmol g^−1^)	k_2_	R^2^
Langmuir	4	100	95.04	0.00328	0.996
			Q_e_^b^	k_1_(h^−1^)	R^2^
Adsorption in different solution pH	4	100	43.1	0.926	0.982
	5	100	25.1	0.765	0.970
	6	100	25.8	0.406	0.959
	7	100	22.1	0.330	0.958
Oxidation in different solution pH	4	100	61.6	0.0014	0.994
	5	100	57.2	0.00083	0.997
	6	100	7.21	0.0017	0.984
	7	100	14.1	0.00042	0.993
Adsorption in different Se(IV) initial concentrations	4	50	50.7	1.325	0.903
	4	100	43.1	0.926	0.982
	4	200	20.2	0.416	0.892
Oxidation in different Se(IV) initial concentrations	4	50	25.8	0.0023	0.992
	4	100	61.6	0.0014	0.994
	4	200	62.1	0.0024	0.994

^a^ Q_max_ (μmol g^−1^) is a constant related to the maximum amount of the adsorption, and k_2_ is the constant related to adsorption affinity. ^b^ Qe is the amount of Se(IV) adsorbed or oxidized at equilibrium, and k^1^ (h^−1^) is the rate constant of the pseudo first-order kinetic equation of the reaction.

**Table 2:** The fitting kinetic parameters of Se(IV) oxidation by δ-MnO_2_ influenced by solution pH, flow rates, initial concentrations, and mineral dosages under stirred-flow condition.

Factors				Kinetic Parameters	
Flow Rate (mL min^−1^)	Initial Se(IV) (μM)	pH	δ-MnO_2_ Dosage (g L^−1^)	K ^a^ (h^−1^)	R^2^
0.5	100	4	10	0.011	0.937
1	100	4	10	0.032	0.940
2	100	4	10	0.035	0.947
1	50	4	10	0.029	0.940
1	100	4	10	0.032	0.940
1	200	4	10	0.025	0.943
1	100	4	10	0.032	0.940
1	100	5	10	0.012	0.822
1	100	6	10	0.00072	0.393
1	100	4	4	0.017	0.973
1	100	4	10	0.032	0.940
1	100	4	20	0.024	0.432

^a^ K is the oxidation rate of Se(IV) on δ-MnO_2_.

**Table 3:** Binding energy and proportions of Mn and Se in the δ-MnO_2_ near-surface reacted with 100 μM of Se(IV).

Sample	Binding Energy/eV	Chemical Form	Area% ^a^			
			0 h	10 h	200 h	700 h
Mn (2p_3/2_)	641.4	Mn(II)	24.02	24.45	24.96	25.08
	641.4	Mn(III)	21.76	23.56	36.76	47.33
	641.8	Mn(IV)	54.22	51.99	38.27	26.59
Se (3p)	164.5~164.7	Se(IV)	0	100	100	100
	170.5~170.7	Se(IV)				
	164.4	Se(VI)	0	0	0	0

^a^ Area% is the atomic percent of Se and Mn on the surface calculated by XPS.

## Data Availability

No data reported.

## Supplementary Materials

The following are available online at https://www.mdpi.com/1660-4601/18/6/2902/s1, Figure S1: The XRD spectra of pristine δ-MnO_2_. Figure S2: SEM of pristine δ-MnO_2_ (1 μm). Figure S3: Se(IV) sorption isotherms on δ-MnO_2_. Figure S4: Se(VI) adsorption on δ-MnO_2_ in batch and stirred-flow experiments. Figure S5: Se 3d spectra of δ-MnO_2_ after reaction with 100 μmol Se(IV) for 0 h, 1 h, 10 h and 700 h.
