# Supplementary material for: Simultaneous Kinetics of Selenite Oxidation and Sorption on δ-MnO2 in Stirred-Flow Reactors
Source: Int J Environ Res Public Health. 2021 Mar 12;18(6):2902. doi: 10.3390/ijerph18062902 (PMC7998768; doi:10.3390/ijerph18062902)
Supplement: Supplementary file 1 [file ijerph-18-02902-s001.pdf]

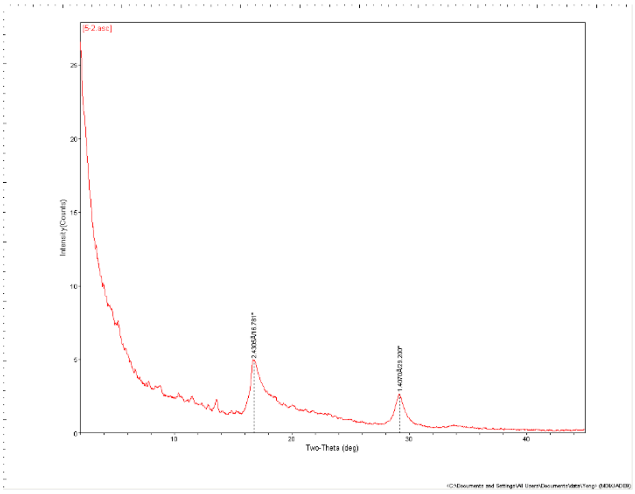

Figure S1. The XRD spectra of pristine δ-MnO2.

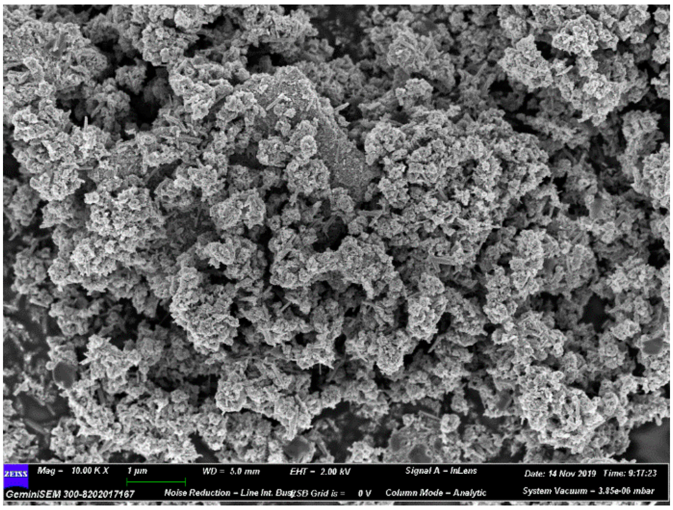

Figure S2. SEM of pristine δ-MnO2 (1μm).

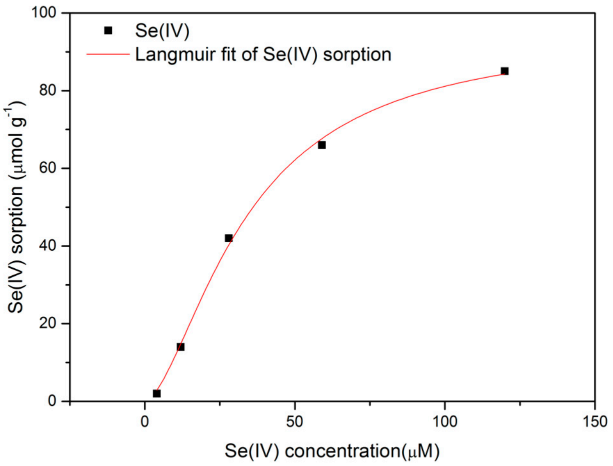

Figure S3. Se (IV) sorption isotherms on δ-MnO2.

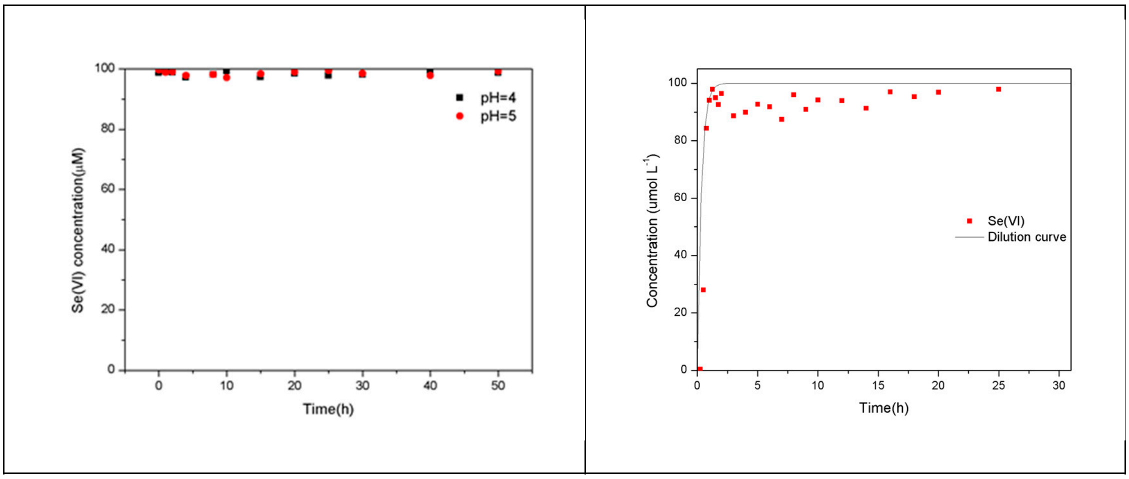

Figure S4. Se (VI) adsorption on δ-MnO2 in batch and stirred-flow experiments.

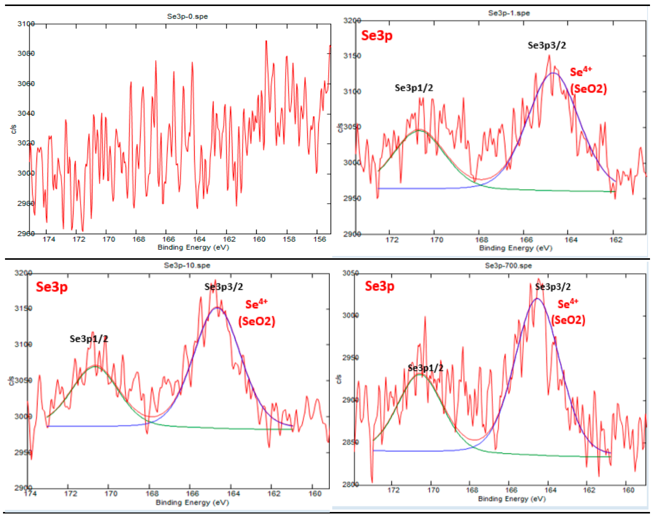

Figure S5. Se 3d spectra of δ-MnO_2 after reaction with 100μmol Se(IV) for 0h, 1h, 10h and 700h.
